# CFD Modeling of HBI/scrap Melting in Industrial EAF and the Impact of Charge Layering on Melting Performance

**DOI:** 10.3390/ma17215139

**Published:** 2024-10-22

**Authors:** Orlando Ugarte, Jianghua Li, Jeff Haeberle, Thomas Frasz, Tyamo Okosun, Chenn Q. Zhou

**Affiliations:** 1 Center for Innovation through Visualization and Simulation (CIVS), Steel Manufacturing Simulation and Visualization Consortium (SMSVC), Purdue University Northwest, Hammond, IN 46323, USA; ougarte@pnw.edu (O.U.); tokosun@pnw.edu (T.O.); 2 Cleveland-Cliffs Research and Innovation Center, Middletown, OH 45005, USA; jianghua.li@clevelandcliffs.com; 3 Cleveland-Cliffs Mansfield Works, Mansfield, OH 44903, USA; jeff.haeberle@clevelandcliffs.com (J.H.); thomas.frasz@clevelandcliffs.com (T.F.)

**Keywords:** electric arc furnace, HBI, CFD, scrap layering, melting, optimization

## Abstract

The melting of scrap and hot briquetted iron (HBI) in an AC electric arc furnace (EAF) is simulated by an advanced 3D computational fluid dynamics (CFD) model that captures the arc heating, the scrap/HBI melting process, and the solid collapse mechanisms. The CFD model is used to simulate a scenario where charge layering and EAF power profiles are provided by a real EAF operation. CFD simulation of the EAF operation shows proper prediction of the charge melting when compared with standard industry practice. Namely, the CFD model predicts a 32.5%/67.5% ratio of solid/liquid steel at the beginning of refining, which approaches the 30%/70% ratio used in standard practice. Based on this prediction, the melting rate in the CFD results differs by 8.3% from actual EAF operation. The impact of charge layering on melting is also investigated. CFD results show that distributing charge material into a greater number of layers in the first bucket (10 layers as compared to 4) enhances the melting rate by 12%. However, including dense material at the bottom of the furnace deteriorates melting performance, reducing the impact of the number of layers of the charge. The CFD platform can be used to optimize the use of HBI/scrap in real EAF operations and to determine best recipe practices.

## 1. Introduction

The electric arc furnace (EAF) is a steelmaking technology that uses electrical and chemical energy for the melting of ferrous scrap and production of steel. The importance of EAFs has increased significantly in recent years worldwide, due in part to the low-carbon emissions associated with EAF operations. The process can produce up to 63% less carbon dioxide emissions than the blast furnace/basic oxygen furnace [1] production route utilized in integrated steelmaking. The need for a reduction in carbon emissions in the iron and steel industry (around 7% of global CO_2_ emissions) has encouraged steelmakers to transition some of their operations to EAF-based production in order to reach carbon reduction goals set by 2050 [1,2].

EAF operation begins with the charging of scrap and/or other iron units. This is followed by pre-heating (in some operations) and melting of the charge. The melting is driven by arc heating (65% of supplied energy in modern EAFs) and burners. After liquid steel is formed, a refining process is completed so that the desired steel composition and temperature are obtained, before tapping of the molten steel. The increasing use of EAF technology can be impacted by the availability of scrap material. EAF operations allow for the use of materials such as pig iron and direct reduced iron (DRI). Among these alternatives, hot briquetted iron (HBI) is a good alternative as it provides a predictable composition (high levels of Fe and low concentration of residual metallic material). HBI is obtained by compacting DRI at a temperature above 650 C. As a result, HBI is more stable and prevents safety issues associated with shipping and handling.

Gonzalez et al. [3] investigated the melting of DRI in an electric arc furnace. The authors applied a Eulerian–Lagrangian model to study the melting of DRI in molten steel. Results showed that melting is enhanced by long arcs, and that the melting is impacted by the feeding process and DRI properties. The study concluded that 50% of melting time corresponded to a frozen shell period, which, in turn, depended on the size of DRI particles. Caffery et al. [4] analyzed the melting of HBI cylinders by using a laboratory induction furnace. Results showed that HBI melts faster than steel due to C and FeO being present in the briquettes. The investigation was complemented by a heat transfer analysis that demonstrated how the carbon content of HBI enhances HBI melting. Namely, carbon reduces the liquidus temperature and increases CO formation, which contributes positively to the melting process.

The technology used in DRI production also has an impact on the melting of DRI. Dutta and Sah [5] discussed direct reduction processes such as rotary kiln and rotary hearth processes (coal-based) and Midrex, HYL, and Purofer processes (gas-based) for DRI production. The study shows that gas-based methods allows for better control of the carbon content of DRI. The authors concluded that DRI quality is improved by using iron ore pellets with a high iron content and low gangue content. Morales et al. [6] proposed a dynamic foaming index (DFI) to control slag foaming in an EAF operating with DRI. The DFI was included in a mathematical model and compared with plant data. The comparison showed that DFI variation along the process resembled the arc distortion trend. Therefore, the DFI was proposed as an indicator of the foaming efficiency in the process, which is a key parameter for process optimization.

Kiasaraei [7] performed experiments of the DRI decarburization process by using a constant-volume pressure increase approach. The melting was carried out by the interaction of DRI pellets with molten slag in the 1500–1600 °C range. The study revealed two stages in the decarburization process. First, carbon reacting with FeO of DRI and, second, carbon reacting with FeO in slag. The carbon content of DRI was found to play key roles in both stages. Kirschen et al. [8] proposed methods to optimize slag operation in order to improve DRI melting in EAF. These suggestions are based on a dynamic mass and energy model of DRI melting. The study analyzed slag data of 16 EAFs, including both DRI and steel scrap charges. The study concluded that an 8–17 kWh/t electric energy demand can be saved by lowering the standard basicity B_2_ (CaO/SiO_2_) from 2 to 1.4–1.7, which lowers the slag mass and FeO loss. The study also discussed how changes in DRI feeding could allow for the longer retention time of DRI fines and a reduction in FeO residuals.

Alameddine and Bowman [9] further analyzed the impact of DRI (or HBI) feeding in the EAF performance. The electric system operation for continuous charging was compared with the charging in buckets. The optimal feed rate was then analyzed by comparing electrical parameters such as fluctuations and harmonics. Conclusions provided guidelines to achieve maximum arc voltage and to enhance EAF performance. Anderson [10] showed a significant improvement in EAF performance when scrap material is partially replaced with DRI. Data demonstrated that 25% DRI in the system could provide a reduction of 46 kWh/ton (9 min of tap-to-tap time). Moreover, when considering 33% DRI, it is possible to reduce this 46 kWh/st by using continuous charge as compared to a two bucket charge. The study also concluded that carbon included in DRI is a more efficient carbon source than charging or injecting carbon into the system.

Ramirez-Argaez et al. [11] studied the impact of DRI on the melting process under conditions seen in EAF. The authors used a mathematical model to simulate a molten pool comprised of steel liquid and slag in an industrial EAF domain. Results were obtained for multiple arc lengths and heat inputs of the electrodes, indicating the impact of arc heating on flow temperature. Additionally, the melting of DRI particles in slag/steel and steel melts was compared, observing a much faster melting rate for steel melts. It was also concluded that melting rate increases as particle size is reduced. Mathematical modeling of EAF operation needs to deal with the complex processes and physics involved, which includes (for instance) electric arc and combustion modeling, as well as multiphase processes. Fathi et al. [12] proposed a model to compute the distribution of the arc heating in EAF operations. Namely, the study proposed a CAM-based algorithm that dropped computational cost by various orders of magnitude as compared with Cassie–Mayr models. The algorithm requires prior knowledge of arc length and current, and was implemented and validated along with a previous integrated methodology [13,14].

The aforementioned research provides good precedents for the investigation of the HBI and scrap melting process. The literature review also reveals the need for investigations in actual EAF conditions, including both scale and operation parameters. Since the EAF environment is hostile to data collection, mathematical modeling acquires great importance as this can provide information needed for process understanding and optimization. In this study, a CFD methodology is used to simulate the melting stage of a real EAF operation. The EAF operation parameters, including furnace geometry, charge recipe, and power profiles are provided by Cleveland-Cliffs. The CFD model adopts the industry parameters as inputs and computes the melting rate and evolution of solid and liquid mass based on conservation equations, without prior knowledge of these phenomena. In addition, the specific impact of carbon reactions (produced in HBI and other materials) on melting is included. The finite volume method (FVM) approach allows for the application of conservation equations throughout the domain, capturing the transient and non-uniform nature of the real EAF operation, which is essential to analyze the melting process and determine the parameters needed for process optimization.

## 2. Methodology

### 2.1. Actual Tap-to-Tap EAF Process

**Figure 1:**
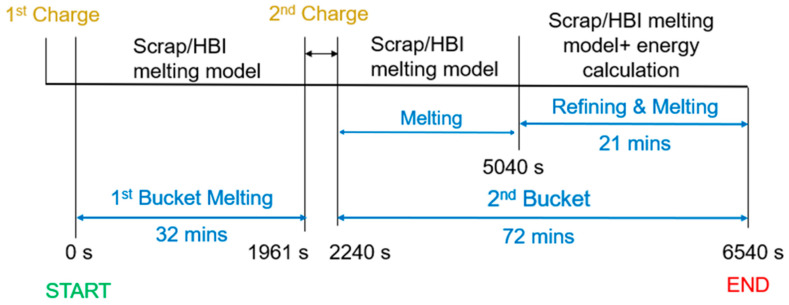
Stages of the tap-to-tap operation carried out by Cleveland-Cliffs [Mansfield, OH, USA].

The CFD simulation applies the operation conditions of a tap-to-tap process performed by Cleveland-Cliffs. A general breakdown of the tap-to-tap operation is illustrated in Figure 1. Specifically, the operation starts with a 1st bucket charge containing 65.7 t of HBI/scrap materials. The melting is initiated by the bore-in of electrodes. After 32 min, the operation is paused and the electrodes withdrawn to charge the 2nd bucket. Here, 66.2 t of additional material is added to the furnace. Then, the melting operation is resumed and continued for another ~47 min. The last 21 min of operation include both melting and refining stages.

The melting of the scrap/HBI material carried out by the arc heating poses a number of modeling challenges. First, proper modeling of AC arc heating is needed, which requires computing of high-frequency phenomena as anode/cathode regions are permanently changing between electrodes and scrap. Additionally, it is necessary to account for proper distribution of the arc heating available at the tip of the electrodes, as this occurs in three ways: radiation, convection, and electron flow mechanisms. The shares (i.e., how much heating is transferred by each mechanism) of the heat transfer mechanisms depend on the arc current and arc length, as well as the electrode position and pit size, which is changing along the process. Another challenge is the interaction between the solid, liquid, and gas phases, as transport phenomena occur between the three phases. The complexities of the EAF modeling also include the presence of multiple reactions, as melting and oxidation reactions occur in parallel to the phenomena mentioned above.

The CFD simulation in this study focuses on the melting of the HBI/scrap charge. Namely, the oxidation reactions produced after 5040 s in Figure 1 are not considered. The scrap melting model used in this study integrates an electric arc model to account for the arc heating delivered by the three AC electrodes, a scrap collapse model to compute the scrap melting and collapse of solid material, and the coherent jet model to compute the chemical energy delivered by burners during the melting stage. In this study, however, the actual EAF operation does not include burners; therefore, the coherent jet model is only used to account for the heating of solid charge produced by the gas heated at the tip of the electrodes. Further details of the models will be shown in Section 2.3.

The simulation conditions are based on the actual heat provided by Cleveland-Cliffs. The data include the dimensions of the actual EAF, the current and voltage of the three electrodes, and the charge recipe of 1st and 2nd buckets. The electrode profiles and charge recipe are used as inputs in the simulation. Next, the pre-processing of these inputs is shown.

### 2.2. Pre-Processing of EAF Operation Data

#### 2.2.1. Electrode Parameters

The raw data provided by Cleveland-Cliffs included arc current and arc voltage for each of the three electrodes. After filtering the data, one of the three electrode profiles is selected and applied to all three phases in the simulation. Based on arc current and voltage information, the arc power is calculated as follows:

(1) $$P_{a}=ui=iR^{2},$$

where the arc resistance is provided by

(2) $$\frac{dR}{dt}=\frac{R}{\tau }\left(1-\frac{ui}{2\pi^{0.5}\sigma^{0.5}l_{a}^{1.5}\sigma_{SB}T_{arc}^{4}R^{-w}}\right).$$

The arc length is calculated as follows:

(3) $$l_{a}=\left(\frac{u-40}{11.5}\right)$$

**Figure 2:**
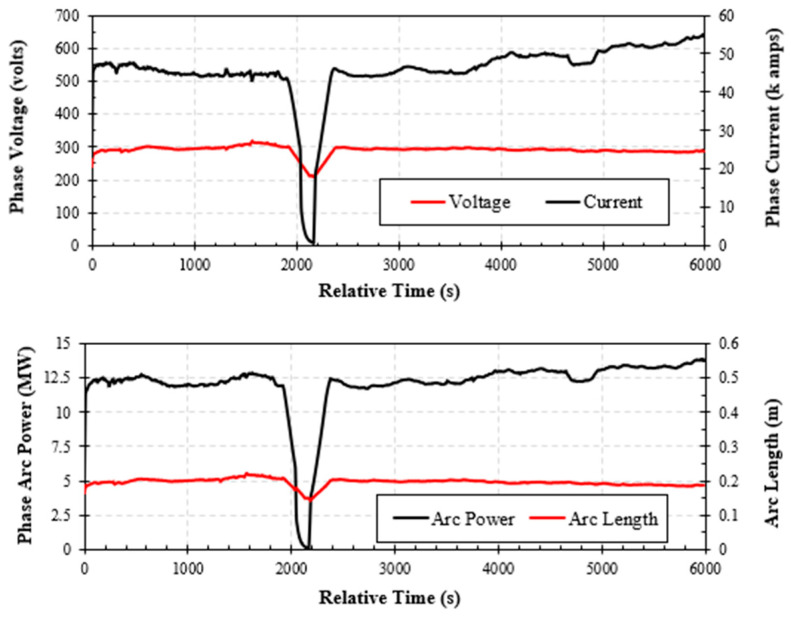
Filtered voltage and current data provided by Cleveland-Cliffs (**top**). Arc power and length applied to CFD simulator (**bottom**).

Figure 2 shows the voltage and current profiles (top) and the arc power and arc length profiles computed in Equations (1) and (2).

#### 2.2.2. Scrap Layering

The recipe used in the actual EAF heat includes multiple layers. The layering materials are listed in Table 1. In the CFD model, the chemistry of the layers is not considered, except for the carbon content (i.e., in HBI) which reacts when oxygen is available and releases additional heating. The impact of specific layers on melting is characterized by its bulk density, which determines its volume fraction. Table 1 shows the volume fraction and mass of each layer.

### 2.3. Integrated EAF-CFD Model

The EAF-CFD solver computes the melting of the HBI/scrap charge driven by the electrical arc heating. As mentioned earlier, the actual EAF operation requires the modeling of multiple phenomena such as arc heating, scrap melting, phase changes, etc. Therefore, the EAF scrap melting model integrates multiple models to compute these processes. These CFD models are the scrap melting model, the electric arc model and the coherent jet model. The scrap melting model computes the melting of the solid phase as it receives the electrical and combustion heating in the EAF. The model computes the solid interaction with liquid and gas phases as well as proper phase change phenomena. The scrap dynamics includes vertical collapse as voids are formed in the lower layers of the charge. Further details of the model implementation can be found in Refs. [15,16,17]. The electric arc model computes the AC arc heating provided by the electrodes during the simulation. This model computes the arc power and length based on the transient arc current and voltage during the process. The arc heating is then distributed in the furnace as radiation, convection, and electron flow mechanisms based on a formulation obtained for real AC-EAFs. Further details of the model implementation and validation can be seen in Refs. [18,19]. A third model developed for the EAF operation is the coherent jet model. This model accounts for the combustion energy supplied to the EAF through coherent jets, which are normally included at the walls of the EAF. The coherent jet model accounts for proper interaction between solid and gas phases, so that even in the case that coherent jets are not included in the operation (as in the heat provided by Cleveland-Cliffs), scrap heating carried by hot gases in the system is properly captured. The implementation and validation of this model has been discussed in detail in Refs. [17,20].

**Figure 3:**
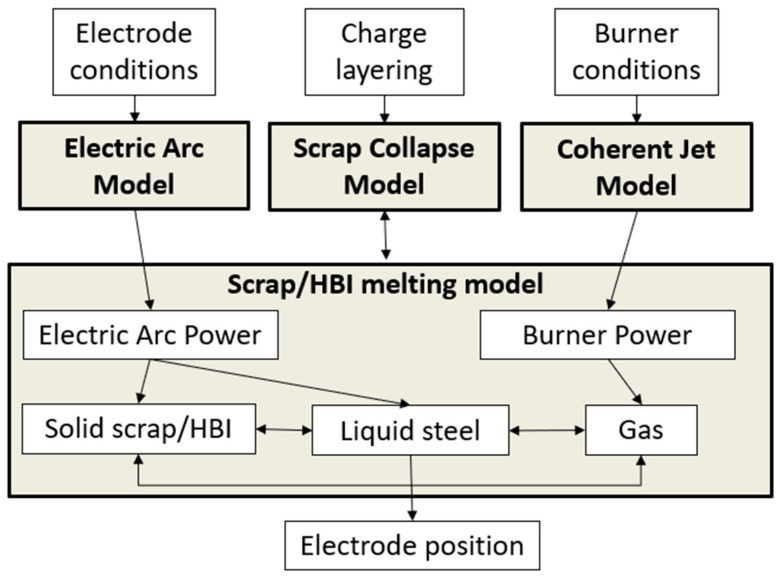
Integration methodology of CFD scrap/HBI melting solver.

The scrap melting, electric arc, and coherent jet models have been integrated in order to compute the actual melting operation in an industry-size EAF. The integration methodology is shown in Figure 3. The solver has been extended to include reactions of carbon present in the charge (i.e., in the HBI layers), which provides additional heating in case oxygen is available. It should be noted that the scrap/HBI layers are characterized by their bulk density, which is used to determine the volume fraction of each layer. The impact of other elements on the melting process is not considered.

Next, a brief description of the finite volume method (FVM) framework is shown. Further details of the integrated CFD solver have been detailed in a prior publication [15].

#### 2.3.1. Scrap Melting Model

##### Fluid Phases

The scrap melting model applies a Eulerian–Eulerian approach to compute the liquid and gas phases. Specifically, conservation equations are solved with source terms accounting for mass and momentum conservation for the interphase interaction forces, and for the momentum induced by arc impingement on the molten bath.

The mass, momentum, and energy conservation equations for gas and liquid phases are given by Equations (4)–(6), respectively:

(4) $$\frac{\partial \left(\alpha_{q}\rho_{q}\right)}{\partial t}+\nabla \cdot \left(\alpha_{q}\rho_{q}\vec{\nu_{q}}\right)={\dot{m}}_{s,mt},$$

(5) $$\frac{\partial \left(\alpha_{q}\rho_{q}\vec{\nu_{q}}\right)}{\partial t}+\nabla \cdot \left(\alpha_{q}\rho_{q}\vec{\nu_{q}}\vec{\nu_{q}}\right)=-\alpha_{q}\nabla p+\nabla \cdot \bar{\bar{\bar{\tau_{q}}}}+{\vec{F}}_{q,df}+P_{l}+\alpha_{q}\rho_{q}\vec{g}$$

(6) $$\frac{\partial \left(\alpha_{q}\rho_{q}E_{q}\right)}{\partial t}+\nabla \cdot \left(\alpha_{q}\vec{\nu_{q}}\left(\rho_{q}E_{q}+p\right)\right)=\nabla \cdot \left(K_{effq}\nabla T_{q}-\sum_{i=1}^{n}h_{ig}{\vec{J}}_{ig}+\bar{\bar{\bar{\tau_{q}}}}\cdot \vec{\nu_{q}}\right)+Q_{q,ht}+Q_{l,arc}$$

The energy conservation equation includes the energy released by combustion reactions, along with the heating of the phases by the arc ($Q_{l,arc}$). In addition, the standard k-ε turbulence model is used to account for turbulence developing between the fluid phases.

##### Solid Phase

The solid phase is considered a porous medium in the scrap melting model. A dual-cell approach is used to compute the mass and energy balance between solid and fluid phases. The mass and energy balance of the solid phase are determined as follows,

(7) $$\frac{d\left(m_{s}\right)}{dt}=-{\dot{m}}_{s,mt}$$

(8) $$\frac{d\left(E_{s}\right)}{dt}=\bar{\bar{Q_{s}}}$$

##### Phase Interactions

The model considers two additional phase interactions, namely, the drag force and energy exchange between the phases. The scrap charge produces a drag force on the fluid, which is characterized by a pressure drop. The drag is modeled by non-Darcian law [21], whereas the solid permeability and inertial resistance are provided by the Ergun equation [22]. The heat transfer between phases is calculated along with force interactions. The coefficient of convective heat transfer from liquid to solid phase is calculated for two regions: above-bath and in-bath conditions [23].

##### Melting/Re-Solidification

The solid phase turns to liquid when the solid temperature is larger than the liquidus temperature ($T_{liquidus}$), and re-solidification occurs when the liquid steel temperature drops below the solidus temperature ($T_{solidus})$. This process is mathematically expressed as

(9) $${\dot{m}}_{s,mt}=\left\{\begin{array}{c} \frac{dm_{s}}{dt}\;\;\;\;\;\;\;\;\left(T_{s}\geq T_{liquidus}\right)\\ -\frac{dm_{l}}{dt}\;\;\;\;\;\left(T_{l}<T_{solidus}\right). \end{array}\right.$$

The additional heat (enthalpy of fusion) involved in melting and re-solidification is modeled by allocating an elevated effective specific heat capacity within the solidus and liquidus temperatures [11]. The effective specific heat capacity $C_{p,eff}$ is calculated as:

(10) $$C_{p,eff}=\left\{\begin{array}{c} C_{p,s}\;\;\;\;\;\;\;\;\;\;\;\;\;\;\;\;\;\;\left(T<T_{solidus}\right)\\ C_{p,s}+\frac{h_{fusion}}{T_{liquidus}-T_{solidus}}\;\;\left(T_{solidus}\leq T\leq T_{liquidus}\right)\\ C_{p,l}\;\;\;\;\;\;\;\;\;\;\;\;\;\;\;\;\;\;\;\;\left(T>T_{liquidus}\right) \end{array}\right.$$

##### Scrap Collapse

The collapse of solid material during melting is included in the EAF solver. However, the complex, random scrap collapse process [24] is simplified as a vertical collapse mechanism. This is achieved by using a ‘stack approach’ where the scrap vertical position is dynamically updated during the simulation due to insufficient support at the lower scrap layers if melting occurs below solid scrap. The method relies on a consistent mesh structure that ensures mass conservation during scrap relocation and descent.

#### 2.3.2. Electric Arc Model

##### Arc Heat Dissipation

The EAF solver assumes the same AC load in all the electrodes. Here, the arc resistance is computed by using the Cassie–Mayr arc model [25]. The arc heating is then transferred by convection, radiation, and electron flow mechanisms at a rate that depends on the instantaneous RMS arc current and arc length. The radiation heat dissipation is computed by using a modified Monte Carlo (MC) statistical method, so that the solid phase that is computed in parallel to the fluid phases can receive this heating. The electrode tip emits a random beam of radiation which is captured by the cell in the path of the beam. Multiple such random beams are computed and the final radiation is the sum of all the received beams. Further details of the model implementation can be found in Ref [18].

##### Electrode Regulation

The solver regulates the electrode position by keeping a constant impedance approach, which maintains the difference between the measured arc voltage and current at zero. The model adjustment is based on the arc length and arc voltage relationship introduced by Billings [26].

##### Arc Impingement

The arc impinges upon the liquid bath after bore-in, which provides additional momentum to the molten steel. This is modeled by including a momentum term $p_{l}$ on the liquid phase in the region beneath the electrode tips. The term $p_{l}$ is given as

(11) $$p_{l}=m_{l}\sqrt{\frac{\rho_{a}v_{a}^{2}}{\rho_{l}}}.$$

##### Carbon Oxidation

The carbon included in charged HBI/DRI reacts with oxygen available in the EAF and produces carbon monoxide (CO). The rate of carbon reaction is based on the work of Maahs et al. [27]. Specifically, the reaction rate depends only upon the temperature of the solid material. It should be noted that the EAF operation in this study does not include burners, and the simulations are focused on the melting stage only. Therefore, it is expected to be minimal oxygen supply and CO formation during the process.

#### 2.3.3. Coherent Jet Model

In this study, the EAF does not include burners, so the coherent jet model is not activated during the simulations. The burner model implementation can be found in a previous work [20]. However, gas temperature increases near the electrode tips, and provides additional heating which should be accounted for. The gas heating is included in gas and solid energy conservation Equations (6) and (8) by a gas–solid heat coefficient [28,29], namely

(12) $$h_{\left(v\right)gs}=\left\{\begin{array}{c} K_{g}\frac{2+1.1{Pr}_{g}^{0.333}{Re}_{gs}^{0.6}}{d_{s}}\;\;\;\;\;\;\;\;\;\;\;\;\left(T_{g}\leq 1373K\right)\\ h_{gs}A_{gs}=\frac{Af\left(\omega \right){\left|\vec{v_{g}}\right|}^{0.9}T_{g}^{0.3}}{d_{s}^{0.75}}\;\;\;\left(T_{g}>1373K\right) \end{array}\right..$$

### 2.4. CFD Case Setup of EAF Operation

#### 2.4.1. CFD Domain and Boundary Conditions

**Figure 4:**
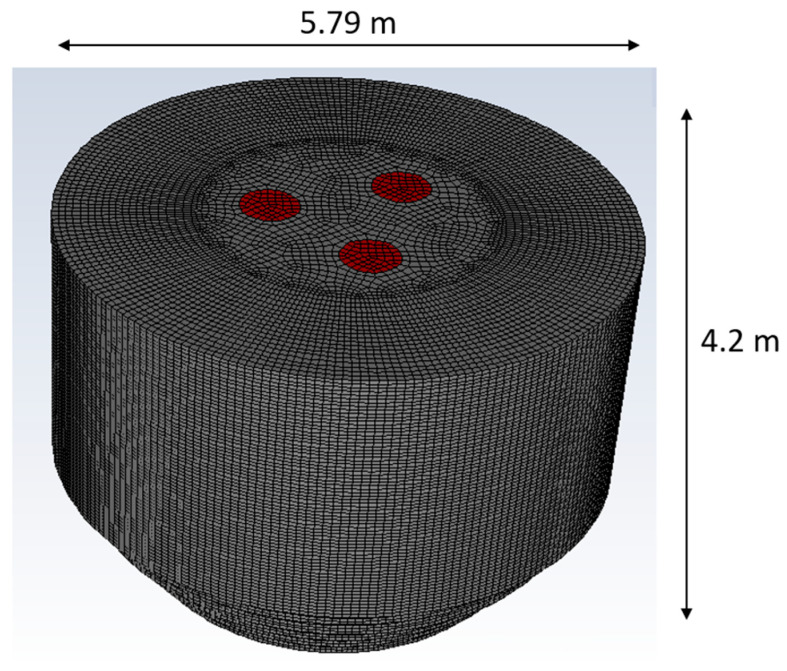
Electric arc furnace computational domain.

A three-dimensional model of the Cleveland-Cliffs EAF has been created in the commercial CFD solver ANSYS Fluent. The basic dimensions of the EAF are shown in Figure 4, which also indicates the electrodes location (red regions on top of the domain). The computational domain for the simulation utilizes a structured grid comprising 603,480 cells. The simulation time is approximately 55,000 CPU hours. The walls are assumed to be adiabatic, non-slip walls. The outlets are approached as pressure outlets where atmospheric pressure is reached (zero-gauge pressure). The electrodes are not physical walls in the CFD setup. Instead, computational cells are identified as electrodes during the simulation, and the position of the electrodes is updated based on the arc length and position of the scrap charge. This approach has demonstrated good comparison of the electrode bore-in process against experimental data in previous studies such as Ref. [15]. The top regions in red are assumed as outlet boundaries. This CFD setup approaches the EAF scenario when the roof is closed after charging of material and the melting of solid charge is in progress.

The CFD-EAF platform is based on a finite volume scheme that employs a pressure-based, transient solver. The solver includes a Eulerian multiphase model, and the viscous model uses a k-epsilon (2 equations) model. The solver accounts for radiation by using the discrete ordinate model. Computations of solid–gas and solid–liquid interactions as well as distribution of arc heating and electrode motion are carried out by in-house codes tailored specifically for the EAF operation at industry scale. The in-house models are integrated into the Fluent platform by using user-defined functions.

#### 2.4.2. Simulation Parameters

Table 2 lists key properties of steel considered in the CFD model during the simulations. The stated assumptions are applied generally to all components of the scrap charge, with the primary differentiating factor between material layers being their bulk density. All other material properties are assumed to match the values in Table 2. It should be noted that the liquidus temperature of HBI material is lower than that of other scrap layers due to its high carbon content. Therefore, the impact of carbon in HBI on melting is twofold, it facilitates melting by lowering the liquidus temperature, and provides additional heating as carbon reacts with available oxygen.

## 3. Validation

**Figure 5:**
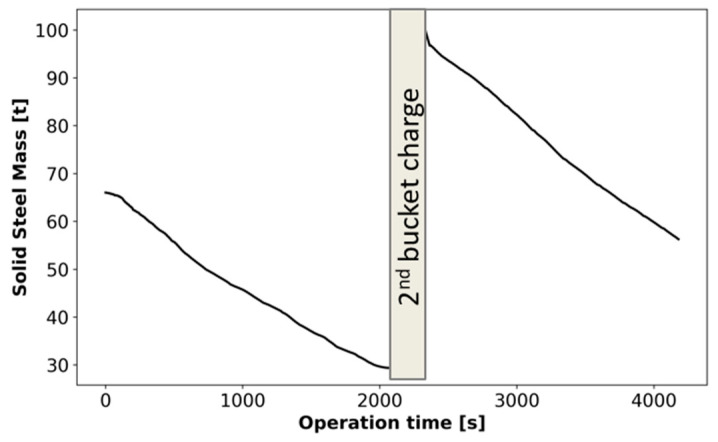
Time evolution of solid mass along melting process.

The baseline simulation is completed based on the EAF dimensions, electrode profiles, and recipe provided by Cleveland-Cliffs (as shown in Figure 2 and Table 1, respectively). The validation of the model is carried out by comparing the amount of liquid and solid steel present at the end of the melting stage, before the beginning of the melting/refining stage. Specifically, industry practice aims for 30% solid/70% liquid steel at the beginning of the refining. The evolution of the solid scrap/HBI in the furnace along the simulation is shown in Figure 5 and listed in Table 3. Namely, scrap/HBI mass drops from 65.7 t at the beginning of the operation to a total of 29.4 t at the end of the first bucket. At 2100 s, 66.2 t of additional material is charged in the second bucket and the melting is resumed. At the end of the melting stage (at t ~5000 s, the moment refining is initiated), CFD results predict 42.8 t of unmelted charge in the furnace. This represents 32.5% of the steel available, and 67.5% of the steel present in liquid form (32.5%/67.5% solid/liquid steel). Therefore, CFD predicts standard industry operation in this scenario properly. Similarly, CFD predicts an averaged melting rate of 18.3 kg/s along the melting process, whereas Cleveland-Cliffs operation would lead to 19.9 kg/s. This represents an 8.3% difference. It should be noted that proper prediction of the melting rate by the CFD model verifies the proper modeling of multiple processes occurring simultaneously, including arc heating, heating distribution, electrode motion, scrap/HBI melting rate, multiphase interaction, and scrap collapse dynamics.

The difference in melting prediction may be partially attributed to the side motion of melting material near the bottom of the furnace. Namely, as melting progresses, solid material collapses due to the formation of voids in the lower regions. This material also slides towards the center of the bottom of the furnace, where it interacts with the molten bath and melts. The side motion is not captured by the CFD solver, which only includes vertical collapse of the solid charge; therefore, the solid falling and melting within the molten bath is underpredicted during the simulation.

## 4. Results

**Figure 6:**
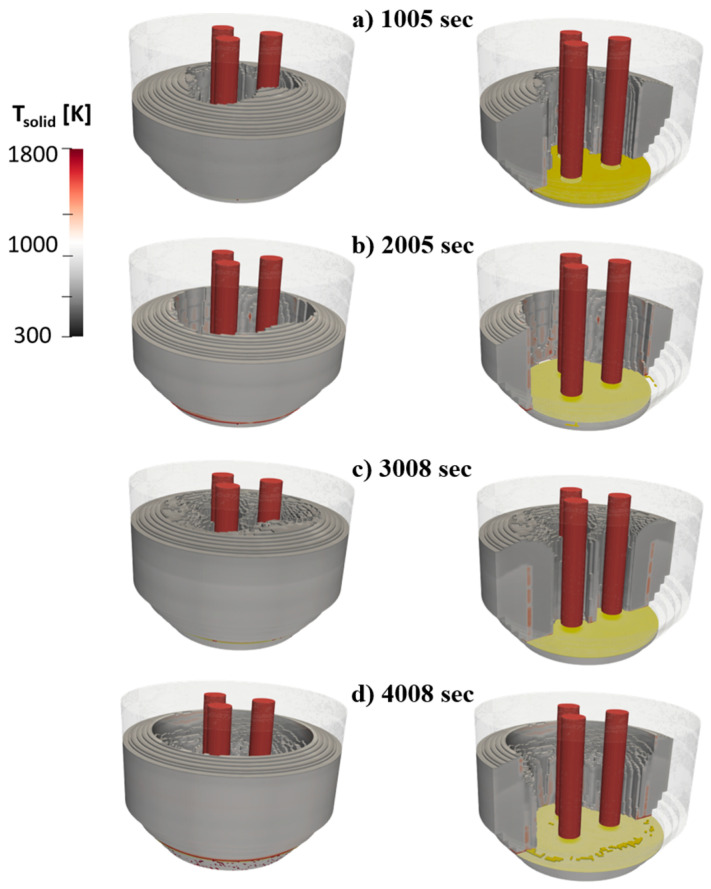
Melting evolution in baseline case at four selected times. (**a**) 1005 s and (**b**) 2005 s correspond to melting of 1st bucket, (**c**) 3008 s and (**d**) 4008 s correspond to instances after charging of 2nd bucket.

Melting of the HBI/scrap charge in the baseline case is shown in Figure 6. Specifically, it shows the electrodes interacting with the charge colored by solid temperature. Results at four separate times through the melting process are shown. The first two timestamps, 1005 and 2005 s, correspond to the first bucket melting (which lasts 2100 s), while the second two timestamps, 3008 and 4008 s, correspond to melting of the second bucket charge. At each instance, a cross-section image through the solid charge is also included that allows the visualization of the molten steel formation and increase in the furnace. In all these instances, it is clear that the melting is produced from the electrodes towards the walls, leading to the increasing of the pit size with time.

**Figure 7:**
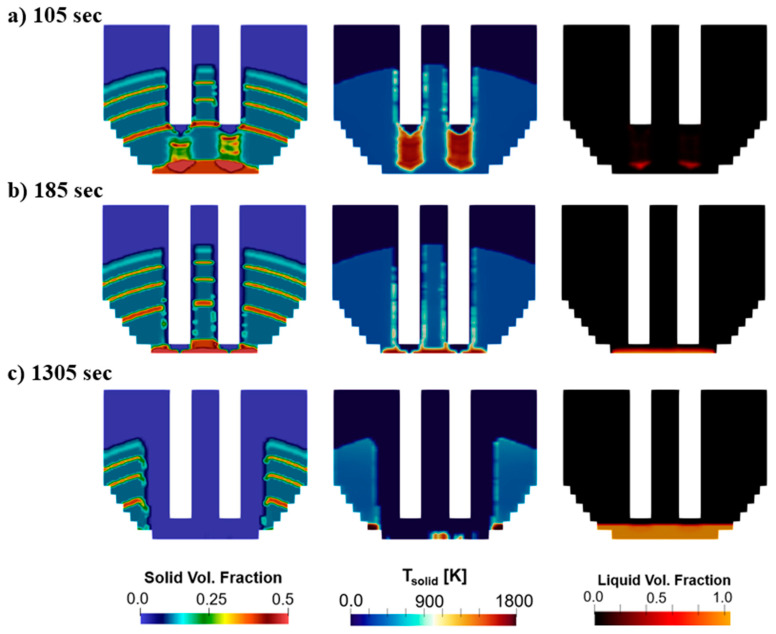
Melting evolution in baseline case along a vertical plane at (**a**) 105 sec, (**b**) 185 s and (**c**) 1305 s.

Further insight into the melting process is observed in Figure 7. Each of the time instances in this example correspond to the first bucket period. These show contours of the solid volume fraction, solid temperature, and liquid volume fraction. The solid volume fraction shows the multiple layers of solid material charged into the furnace, as different materials have varying bulk densities and volume fractions. The solid temperature contours show how the solid charge heats at the tip of the electrodes leading to melting. The liquid volume fraction confirms this observation by showing how the molten steel produced at the tip of the electrodes drips to the bottom of the furnace (right side of Figure 7a). In Figure 7b, the bore-in process is completed and the pit size starts increasing, which is also seen in Figure 7c.

**Figure 8:**
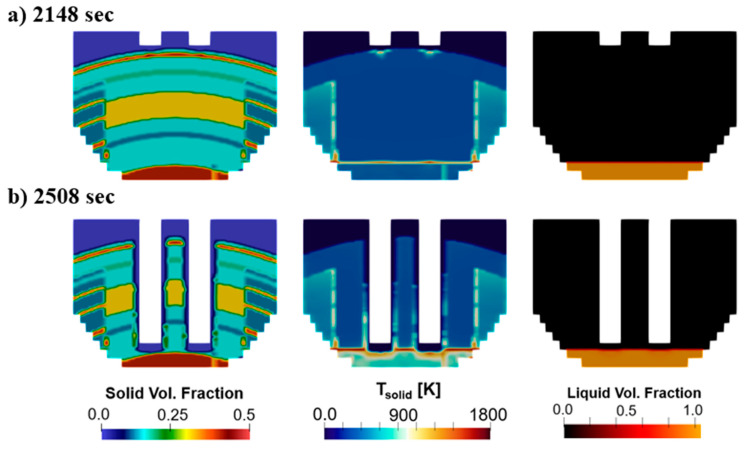
Melting evolution in baseline case along a vertical plane at (**a**) 2148 s and (**b**) 2508 s.

**Figure 9:**
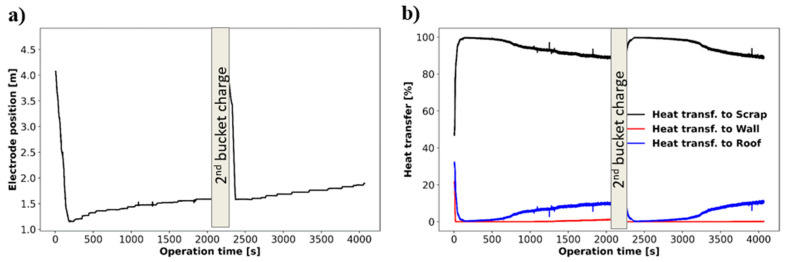
Electrode position (**a**) and heating distributions (**b**) along 1st and 2nd bucket operation.

Figure 8 shows the melting after charging of the second bucket. Figure 8a shows the beginning of the process and Figure 8b shows the end of the bore-in stage. The solid volume fraction shows the new layering charged in the second bucket, which is added on top of the molten steel formed and the unmelted material from the first bucket operation. The solid temperature contours show that the charge is hotter at the walls of the pit formed before the charge of the second bucket, as well as at the molten steel surface. Figure 9 shows the electrode position as well as the heating distribution along the process. Specifically, Figure 9a shows the bore-in of the electrodes after the charge of the first (at t = 0 s) and second buckets (at t = 2100 s). Electrode bore-in for the first bucket is accomplished in 184 s compared to 198 s in the second bucket. The longer time required after the second bucket charge corresponds to the larger solid mass present in the furnace at that moment, which includes the remaining unmelted material from the first bucket. Figure 9b indicates how the total heating provided by the electrodes is distributed to the scrap, walls, and roof of the furnace. In this scenario, almost 100% of the electrical heating available at the tip of the electrodes is transferred to the scrap shortly after the bore-in is initiated. The amount of energy transferred to the scrap declines over time as the pit size increases and energy is lost to the roof. The heating lost to the walls remains low throughout the process. This repeats after the charge of the second bucket where the percentage of heat transferred to the scrap is larger than 90% until ~3300 s.

## 5. Discussion

This section compares the baseline case discussed earlier with a new case, where the charge recipe is modified. Namely, a new scenario is obtained by reducing the number of layers of the original recipe while maintaining the total weight and volume of the original charge. This is achieved by merging material with similar bulk densities (hence, similar volume fraction) into condensed layering, reducing the number of layers from 14 (10 volume-fraction layers) to 4 in the first bucket, and from 13 (10 volume-fraction layers) to 6 in the second bucket. The new ‘compacted’ scenario is described in Table 4. It should be noted that the recipe of the second bucket includes lime and chrome alloy materials. These materials have different properties to most metallic materials. In this study, properties of the solid layers are assumed the same, except for the bulk density. This simplification deteriorates when non-metallic materials are included in the charge. However, it is possible to determine the impact of bulk density and layering array on melting as it will be shown next, while the impact of the specific thermal conductivity of non-metallic materials is not considered.

**Figure 10:**
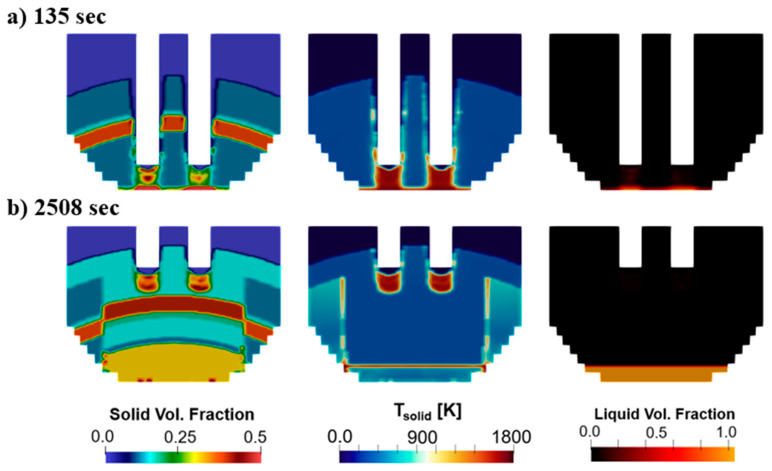
Melting process in compacted layering case at (**a**) 135 s and (**b**) 2508 s.

The melting of solid charge in the compacted scenario is shown in Figure 10. This shows the bore-in in the first bucket (Figure 10a) and the beginning of the melting of the second bucket (Figure 10b). The modified layers are shown in the solid volume fraction contours, where the denser material HBI (reddish layer) is located at the center in the two buckets. In the second bucket, a large region with medium bulk density is located at the bottom layer.

**Figure 11:**
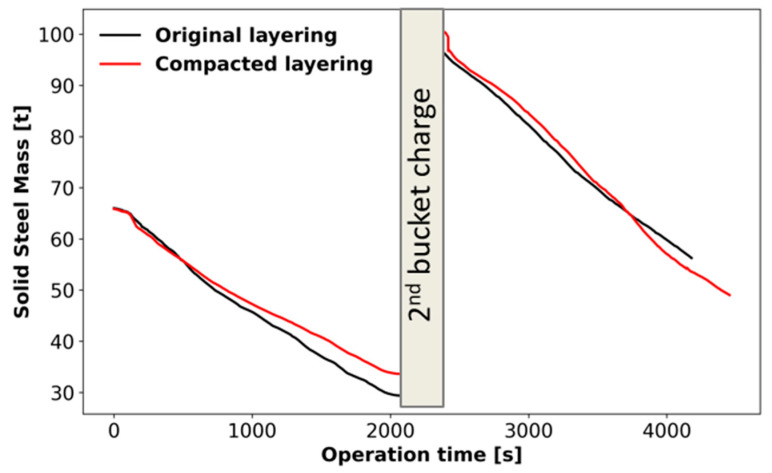
Comparison of solid mass evolution for original and compacted charge layering.

The impact of charge layering is seen in Figure 11, where the solid mass evolution is compared for the original and compacted scenarios. In the melting of the first bucket, the original layering leads to a larger melted mass. By the end of this period, the original layering melts 4.3 t more charge than the compacted layering. In the melting of the second bucket, however, the trend changes, and the compacted layer leads to 2.8 t more melted charge.

**Figure 12:**
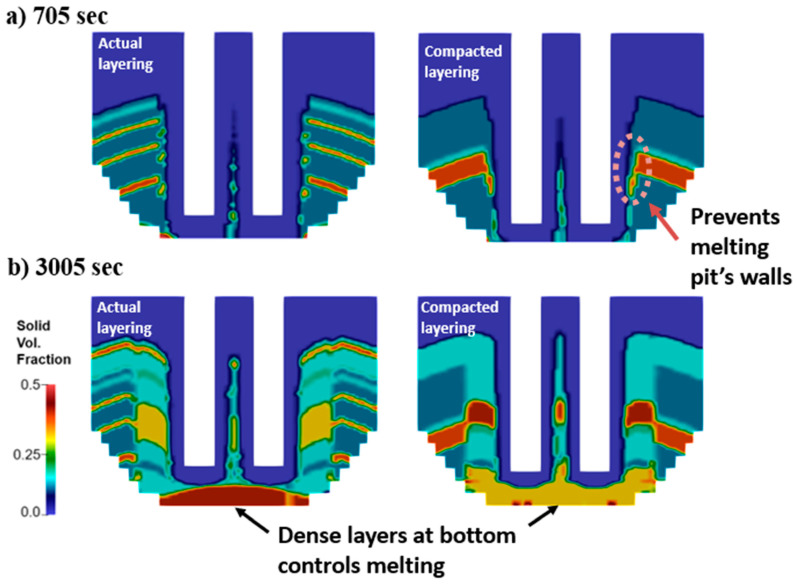
Impact of layering on melting performance at (**a**) 705 s and (**b**) 3005 s.

The melting performance is modified by the number of layers charged in the EAF, which is the only difference between the original and compacted scenarios. Figure 12 shows the impact of layering on melting at the first bucket (Figure 12a) and second bucket (Figure 12b). At t = 705 s, the bore-in is completed for both scenarios and the melting leads to the collapse of the pit walls. In the compacted case, however, a large portion of the wall containing dense material is collapsing and shields the lighter material behind it from radiation coming from the electric arc (Figure 12a, right). Since the dense material melts slower than light material, this shielding reduces the melting rate, leading to the differences observed in Figure 11 starting at t = ~700 s. In the melting of the second bucket (Figure 12b), dense material is located at the center of the furnaces for both scenarios. However, both cases contain dense material at the bottom as well. These dense layer regions deteriorate melting efficiency for both scenarios and it causes the similar solid mass evolution seen in Figure 11 after 2200 s. In fact, the bottom layer in the compacted scenario is less dense than in the original case, and a slightly faster melting rate is obtained.

**Figure 13:**
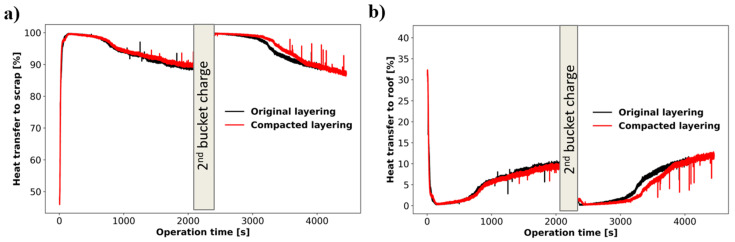
Impact of charge layering on electrical heating distribution (**a**) percentage of electrical heating transferred to scrap and (**b**) percentage of electrical heating transferred to roof for both scenarios.

The impact of layering is also reflected in the heating efficiency produced in the operation. Figure 13 shows similar scrap heating and heating losses to the roof in the first bucket. This is expected as the arc heating is mostly being transferred to the solid charge in both scenarios after bore-in. The difference in scrap heating efficiency is significant after 3000 s of operation (during melting of the second bucket) where the compacted scenario leads to a more efficient scrap heating. In the first bucket, the efficiency of energy transferred to scrap is similar in both scenarios, but the original layering is able to melt a larger mass of solid material. As seen in Figure 13a, the dense layers falling from the pit walls in the compacted case consumes the thermal energy available, preventing further melting of light scrap behind it. In the second bucket, the dense layer of material at the bottom reduces heat transfer efficiency in both cases, but the bulk density of the bottom layer in the compacted recipe is 0.30, which is lower than the 0.37 bulk density considered in the original recipe. This results in an increased heating efficiency of compacted layering near the end of the operation.

## 6. Conclusions

In this study, a CFD platform is applied to study the melting of scrap/HBI charge in a real EAF operation. The EAF operation conditions are provided by Cleveland-Cliffs. Specifically, the EAF heat includes 121.9 tons of HBI/scrap material distributed in 14 layers in the first bucket (65.7 tons) and 13 layers in the second bucket (66.2 tons). The EAF does not include burners, and all the thermal energy is supplied by AC electrodes. The charge recipe, electrode voltage, and current provided by Cleveland-Cliffs are applied to a baseline simulation.

The baseline simulation shows that the melting onset occurs at the tip of the electrodes. Namely, the electrodes generate a pit in the center of the furnace that increases as melting progresses. The material at the walls of the pit falls and melts in the molten bath. Baseline simulation predicts 32.5% of steel in the furnace is solid at the time of refining, whereas 67.5% is in liquid phase. This prediction agrees with industry practice that aims for a 30%/70% ratio at this time.

The impact of the charge layering on melting performance is studied by reducing the number of layers in the first and second buckets. This is achieved by merging the same material types into single layers. By doing this, the first bucket layering reduced from 14 (10 volume-fraction layers) to 4 layers, and the second bucket layering reduced from 13 (10 volume-fraction layers) to 6 layers. Comparison of original and compacted cases shows that the larger number of layers in the first bucket leads to a 12% higher melting rate in the original case. In the second bucket, however, the melting rate is similar in both cases due to the presence of dense material in the bottom of the furnace, which deteriorates the melting process for both original and compacted scenarios.

Analysis of the energy distribution shows that more than 90% of the electrical energy is transferred to the scrap during the operation, for both original and compacted layering. In both cases, the second bucket charge is produced when the energy lost to the walls and roof reach 10% combined.

## Figures and Tables

**Table 1:** Scrap layering for both 1st and 2nd charge.

Layer	Type	Volume Frac.	Weight [kg]
1	40916 scrap	0.107	2494.8
2	#1 bushels	0.139	3628.7
3	fragmented scrap	0.107	3175.1
4	400 grade mixed reclaim	0.107	2268.0
5	HBI	0.374	6327.6
6	40916 scrap	0.107	2041.2
7	409 home scrap	0.107	6803.9
8	HBI	0.374	6441.0
9	fragmented scrap	0.107	2698.9
10	40916 scrap	0.107	7144.1
11	HBI	0.374	7325.5
12	fragmented scrap	0.107	4535.9
13	409 converter scrap	0.107	5420.4
14	HBI	0.374	5397.7
	Total 1st bucket		65,702.8
**Layer**	**Type**	**Volume Frac.**	**Weight [kg]**
15	40916 scrap	0.107	2676.2
16	HBI	0.374	6418.3
17	40916 scrap	0.107	1474.2
18	fragmented scrap	0.107	8527.5
19	dolomitic lime	0.171	2449.4
20	EAF lime	0.117	5715.3
21	charge chrome 55	0.267	9071.8
22	charge chrome 55	0.267	9298.6
23	400 grade mixed reclaim	0.107	4545.0
24	EAF 75% FeSi lump	0.085	1134.0
25	fragmented scrap	0.107	6282.2
26	40916 scrap	0.107	2268.0
27	HBI	0.374	6373.0
	Total 2nd bucket		66,233.5

**Table 2:** Material properties of steel considered in tap-to-tap simulation.

Parameter	Symbol	Value	Unit
Steel Density	ρ	7500	kg/m^3^
Solidus Temperature	T_solidus_	1600	K
Liquidus Temperature	T_liquidus_	1809	K
HBI Liquidus Temperature	T_liquidus, HBI_	1798.1	K
Solid Steel Specific Heat	C_p,s_	400	j/kg-K
Liquid Steel Specific Heat	C_p,l_	696.4	j/kg-K

**Table 3:** Mass evolution of solid charge in baseline case.

Stage	Scrap/HBI [t]
Initial 1st bucket	65.7
End 1st bucket	29.4
Initial 2nd bucket	95.6
Expected at refining	42.8

**Table 4:** Layering in 1st and 2nd bucket charges of ‘compacted’ scenario.

Layer	Type	Volume Frac.	Weight [kg]
1	40916 scrap	0.107	21,182.0
	40916 scrap		
	409 home scrap		
	fragmented scrap		
	40916 scrap		
2	#1 bushels	0.139	3628.7
3	HBI	0.374	25,491.0
4	fragmented scrap	0.107	15,399.0
	400 grade mixed reclaim		
	fragmented scrap		
	409 converter scrap		
	Total 1st bucket		65,700.7
**Layer**	**Type**	**Volume Frac.**	**Weight [kg]**
1	40916 scrap	0.143	25,773.1
	40916 scrap		
	fragmented scrap		
	400 grade mixed reclaim		
	fragmented scrap		
	40916 scrap		
2	HBI	0.400	12,791.3
3	dolomitic lime	0.171	2449.4
4	EAF lime	0.143	5715.3
5	EAF 75% FeSi lump	0.107	1134.0
6	charge chrome 55	0.300	18,370.4
	Total 2nd bucket		66,233.4

## Data Availability

The data presented in this study is available on request from the corresponding author. Operation conditions considered in this study are proprietary to Industry partner. Data release needs to be authorized by the Steel Manufacturing Simulation and Visualization Consortium.

## Nomenclature

q	Phase Subscript (l for Liquid, g for Gas, and s for Solid)
α	Volume fraction
ρ	density
ν	velocity
p	pressure
${\dot{m}}_{s,mt}$	Mass transfer rate between solid and liquid
$P_{l}$	Momentum transfer
h	Heat transfer coefficient
$Q_{q,ht}$	Interphase heat transfer
$Q_{l,arc}$	Arc heat delivery to the liquid steel phase
$m_{s}$	Solid mass
$m_{l}$	Liquid mass
E	Fluid energy
$E_{s}$	Solid energy
t	Flow time
u	Phase voltage
i	Phase current
k	Turbulence kinetic energy
T	Temperature
Re	Reynolds number
$\bar{\bar{Q_{s}}}$	Volumetric energy exchange with fluid phases
$\bar{\bar{\bar{\tau_{q}}}}$	Viscous stress
$T_{liquidus}$	Liquidus temperature
$T_{solidus}$	Solidus temperature
$h_{fusion}$	Fusion latent heat
$C_{p}$	Specific heat capacity
ε	Turbulent dissipation rate
Pr	Prandtl number
d	Characteristic diameter
$\rho_{a}$	Average AC arc density
$v_{a}$	Average AC arc velocity
$C_{T}$	A function of the gas temperature and velocity field
$h_{\left(v\right)gs}$	(Volumetric) Heat transfer coefficients
A, $f\left(\omega \right)$	Material-dependent coefficients
$l_{a}$	Arc length
$\sigma_{SB}$	Stefan–Boltzmann constant
$\vec{J}$	Species mass diffusion
R	Arc resistance
σ	Ionized air’s specific conductivity
